# *Cornus controversa* Leaf and Stem Extract Attenuates LPS-Induced Inflammatory Responses in RAW 264.7 Macrophages via NF-κB/MAPK Inhibition and AMPK Activation

**DOI:** 10.4014/jmb.2509.09052

**Published:** 2025-11-26

**Authors:** Seo Young Choi, Mi Song Shin, Hong-Joo Son, Seon Beom Kim, Parkyong Song, Kwang Min Lee

**Affiliations:** 1 Department of Life Science and Environmental Biochemistry, Pusan National University, Miryang 50463, Republic of Korea; 2 Department of Food Science and Technology, Pusan National University, Miryang 50463, Republic of Korea; 3 Department of Convergence Medicine, Pusan National University School of Medicine, Yangsan 50612, Republic of Korea

**Keywords:** *Cornus controversa* extract, inflammation, lipopolysaccharide (LPS), NF-κB; MAPK, AMPK

## Abstract

Inflammation is a fundamental immune response that protects the host against infection and tissue injury. However, it can also contribute to the pathogenesis of various chronic diseases. In this study, we investigated the anti-inflammatory effects of *Cornus controversa* leaf and stem (CC-LS) extract in RAW 264.7 macrophages as well as the underlying mechanisms. Our results showed that CC-LS attenuated lipopolysaccharide (LPS)-induced inflammatory responses in RAW 264.7 macrophages in a dose-dependent manner without cytotoxicity even at high concentrations. Specifically, CC-LS significantly suppressed nitric oxide production and downregulated the expression of inducible nitric oxide synthase and cyclooxygenase-2 in the LPS-stimulated cells. It also attenuated intracellular reactive oxygen species accumulation, inhibited NF-κB p65 phosphorylation, and downregulated the expression of pro-inflammatory cytokines, including tumor necrosis factor-α, interleukin-6, and interleukin-1β. Moreover, it inhibited the phosphorylation of mitogen-activated protein kinases, ERK, JNK, and p38, while promoting the activation of AMP-activated protein kinase (AMPK) and its downstream substrate, acetyl-CoA carboxylase. These findings indicate that CC-LS exerts potent anti-inflammatory effects in macrophages by targeting the NF-κB, MAPK, and AMPK signaling pathways, suggesting its potential as a natural source for developing anti-inflammatory therapeutic agents.

## Introduction

Inflammation is an essential innate immune response triggered by various harmful stimuli, including pathogens, toxins, and tissue injury [1, 2]. Although it plays a crucial role in host defense and in maintaining homeostasis, excessive or prolonged inflammatory responses are strongly associated with the pathogenesis of various acute and chronic diseases, including arthritis, atherosclerosis, cardiovascular disease, and cancer [2-5]. Thus, regulation of aberrant inflammatory signaling has been recognized as a key strategy for preventing and treating inflammatory disorders.

Macrophages, which are key effector cells in inflammation and immunity, release a wide array of pro-inflammatory mediators (*e.g.*, nitric oxide (NO) and prostaglandin E2 (PGE2)), enzymes (*e.g.*, inducible nitric oxide synthase (iNOS) and cyclooxygenase-2 (COX-2)), and cytokines (*e.g.*, tumor necrosis factor-α (TNF-α), interleukin-1β (IL-1β), and interleukin-6 (IL-6)) [6-8]. Further, lipopolysaccharide (LPS), a component of the outer membrane of gram-negative bacteria that has been sufficiently characterized, activates macrophages via Toll-like receptor 4 (TLR4) [9]. Specifically, LPS stimulation activates intracellular signaling cascades, including nuclear factor-κB (NF-κB) and mitogen-activated protein kinases (MAPKs), which in turn drive the transcription of pro-inflammatory genes as well as the release of inflammatory mediators [10, 11]. Therefore, LPS-stimulated RAW 264.7 macrophages have been extensively used as an established *in vitro* model for identifying potential anti-inflammatory agents and elucidating their underlying mechanisms.

On the one hand, among the signaling pathways that govern macrophage responses to inflammation, NF-κB functions as a key transcription factor that regulates the expression of multiple inflammatory mediators and cytokines [10], and MAPKs, including ERK, JNK, and p38, play important roles in exacerbating inflammatory responses and stabilizing cytokine gene expression [12, 13]. On the other hand, AMP-activated protein kinase (AMPK), primarily known as a master regulator of cellular energy homeostasis, has emerged in recent years as an important negative regulator of inflammation [14].

*Cornus controversa* Hemsl., a member of the Cornaceae family, is extensively distributed across East Asia and has a long history of use as a traditional medicinal plant [15-17]. Also known as the wedding cake tree, this deciduous species is distinguished by its characteristic tiered branching morphology, which makes it suitable for ornamental purposes. While other *Cornus* species, such as *Cornus officinalis*, have been investigated for their pharmacological properties [18], the biological effects of *Cornus controversa* remain poorly understood, and to the best of our knowledge, there is no systematic study on the anti-inflammatory effects of its leaf and stem extract in macrophages. Therefore, in this study, we aimed to investigate the anti-inflammatory effects of *Cornus controversa* leaf and stem (CC-LS) extract in LPS-stimulated RAW 264.7 macrophages and elucidate the underlying mechanisms. The findings of this study may provide evidence for the use of *Cornus controversa* in the development of anti-inflammatory drugs.

## Materials and Methods

### Preparation of CC-LS Extract

The plant extract (KPM033-091) used in this study was obtained from the Natural Product Central Bank at the Korea Research Institute of Bioscience and Biotechnology (Republic of Korea). The extract was prepared as follows. *Cornus controversa* leaves and stem were collected from Dunnae-myeon, Hoengseong-gun, Gangwon-do, Republic of Korea in 2008, air-dried under shade, and powdered. Thereafter, 1 L of methanol (99.9%, HPLC grade) was added to 80 g of the leaf and stem powder and extracted via 30 cycles (40 KHz, 1,500 W, 15 min ultrasonication, with standing for 120 min per cycle) at room temperature using an ultrasonic extractor (SDN-900H, SD-Ultrasonic Co., Ltd., Republic of Korea). After filtering using Qualitative Filter No.100 (Hyundai Micro Co., Ltd., Republic of Korea) and drying under reduced pressure, the CC-LS extract (11.37 g) was obtained.

### Cell Culture

The mouse macrophage cell line RAW 264.7 was provided by Professor Myunghoo Kim (Seoul National University, Republic of Korea). The cell line was cultured in Dulbecco’s modified Eagle’s medium (DMEM; Welgene Inc., Republic of Korea) supplemented with 10% heat-inactivated fetal bovine serum (Cytiva, USA), penicillin (100 units/ml), and streptomycin sulfate (100 μg/ml), with the medium maintained at 37°C in a humidified atmosphere containing 5% CO_2_. Thereafter, the cell line was pretreated with various concentrations of CC-LS for 24 h followed by washing twice with phosphate-buffered saline (PBS), and subsequent exposure to 1 μg/ml LPS (L3129, Sigma–Aldrich, USA) for 24 h.

### Cell Viability Assay

Cell viability after the CC-LS treatment was determined using the water-soluble tetrazolium salt 1 (WST-1; EZ-CyTox, Republic of Korea) assay. In brief, RAW 264.7 macrophages were seeded into 96-well plates at a density of 2 × 10^4^ cells/well, incubated in DMEM for 24 h. Next, the cells were treated with the indicated concentration of CC-LS for 24 h, and cell viability was measured via the WST-1 assay according to the manufacturer’s instructions. Specifically, the WST-1 solution was added to each well at 10% of the total volume of each well followed by incubation for 1 h, after which absorbance was measured at 450 nm using a microplate reader (LTek, Republic of Korea). The optical density of the control cells was assumed to represent 100% cell viability.

### Measurement of NO Production

RAW 264.7 macrophages were seeded into 96-well plates at a density of 2 × 10^4^ cells/well and allowed to adhere overnight. Thereafter, the cells were pretreated with various concentrations of CC-LS for 2 h and then stimulated with LPS (1 μg/ml) for 24 h. Next, supernatants were collected from each well and centrifuged, and the nitrite concentration in the resulting supernatant sample was measured as an indicator of NO production using the NO Plus Detection kit (iNtRON Biotechnology, Republic of Korea) in accordance with the manufacturer’s instructions.

### Measurement of Reactive Oxygen Species (ROS) Production

Intracellular ROS accumulation in RAW 264.7 macrophages was quantified via 2',7'-dichlorodihydrofluorescein diacetate (DCF-DA) staining. In brief, cells were seeded in 6-well plates at a density of 1 × 10^5^ cells/ml. Following LPS stimulation, the cells were incubated with 50 μM DCF-DA (D6883, Sigma–Aldrich) and 10 μg/ml Hoechst 33342 (H3520, Sigma–Aldrich) for 30 min at 37°C in the dark. Finally, the medium was removed, and the cells were washed twice with PBS. ROS levels, indicated by DCF fluorescence, were determined via fluorescence microscopy (Nikon, Japan).

### Immunoblot Analysis

Proteins in RAW 264.7 macrophages were separated via sodium dodecyl sulfate-polyacrylamide gel electrophoresis (SDS-PAGE) and blotted onto nitrocellulose (NC) membranes using 3% bovine serum albumin in Tris-buffered saline with Tween 20 (137 mM NaCl, 20 mM Tris-Cl, pH 7.6, 0.1% Tween 20). The resulting blots were then incubated with various primary antibodies, including anti-iNOS, anti-COX-2, anti-IL-1β, anti-TNF-α, anti-IL-6, anti-phospho-p44/42 MAPK (ERK1/2), anti-p38 MAPK, anti-phospho-p38 MAPK, anti-JNK, anti-phospho-JNK, anti-nuclear factor kappa-light-chain-enhancer of activated B cells (NF-κB), anti-phospho-NF-κB p65, anti-phospho-AMPKα, anti-ACC, and anti-phospho-ACC all obtained from Cell Signaling Technology (USA), as well as anti-MAPK (ERK1/2; R&D Systems Inc., USA), anti-AMPKα (Invitrogen, USA), anti-GAPDH (Abfrontier, Republic of Korea), and anti-tubulin (Sigma-Aldrich). The blots were subsequently incubated with secondary antibodies (anti-rabbit horseradish peroxidase-conjugate or anti-mouse horseradish peroxidase-conjugate obtained from Santa Cruz Biotechnology (USA). Finally, the resulting protein bands were observed using an enhanced chemiluminescence detection system (Amersham Pharmacia, USA). Further, images of the western blot bands were acquired using the ImageQuantTM 500 system (Cytiva), and densitometric analysis was performed using ImageJ software (NIH, USA).

### Statistical Analysis

All statistical analyses were performed using Origin Software v8.0 (OriginLab Corporation, USA). Significant differences between groups were determined using one-way analysis of variance (ANOVA), followed by Tukey’s post-hoc test for multiple comparisons. Data based on at least three independent experiments were presented as the mean ± standard error of the mean (SEM), and statistical significance was set at *P* < 0.05.

## Results

### CC-LS Suppressed NO Production in LPS-Stimulated Macrophages via iNOS and COX-2 Downregulation

**Fig. 1:**
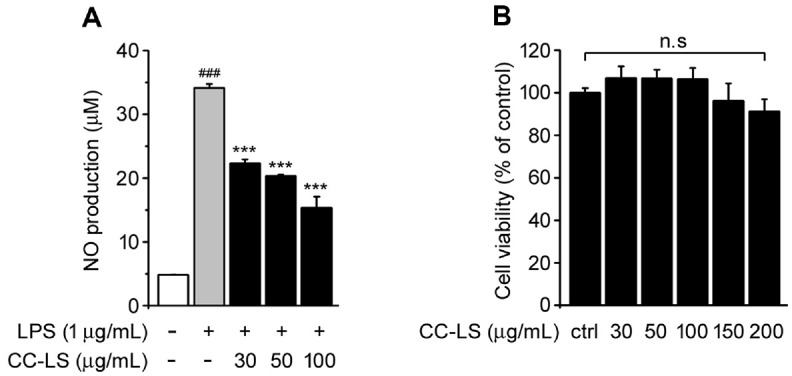
Effects of CC-LS on NO production and viability of Raw 264.7 macrophages. (**A**) Nitric oxide production measured using the Griess reaction. (**B**) Cell viability evaluated via the WST-1 assay. The values in the bar graphs represent the mean ± SEM of at least three independent experiments. ^###^*P* < 0.005, significant difference relative to the control group; ***P < 0.005, significant difference relative to the LPS group.

NO is a central mediator of the macrophage inflammatory response, and its overproduction is a hallmark of LPS-induced activation [19, 20]. To determine whether CC-LS extract modulates this response, nitrite accumulation, an indicator of NO production, was quantified. LPS-treated RAW 264.7 cells showed significantly higher nitrite concentrations than the untreated control cells. However, CC-LS pretreatment significantly inhibited nitrite accumulation in a concentration-dependent manner (Fig. 1A). Additionally, the results of cell viability assays indicated that CC-LS did not exert any cytotoxic effects on the macrophages even at higher concentrations (30–200 μg/ml) (Fig. 1B). These findings indicate that the inhibitory effect of CC-LS on NO generation is independent of any decline in cell viability and may be indicative of specific anti-inflammatory activity.

**Fig. 2:**
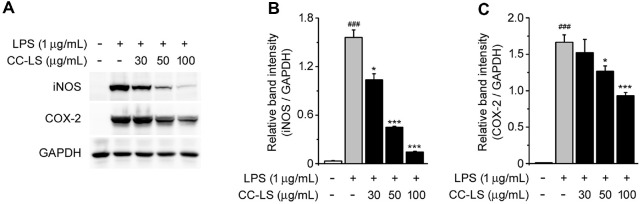
Effects of CC-LS on iNOS and COX-2 expression in LPS-stimulated RAW 264.7 cells. (**A**) Western blot analysis of iNOS and COX-2 expression, with GAPDH as the loading control. (**B**) Relative band intensity of iNOS expression quantified via densitometric analysis and normalized to the expression level of GAPDH. (**C**) Relative band intensity of COX-2 expression quantified via densitometric analysis and normalized to the expression level of GAPDH. The values in the bar graphs represent the mean ± SEM for at least three independent experiments. ^###^*P* < 0.005, significant difference relative to the control group; *P < 0.05, **P < 0.01, ***P < 0.005, significant differences relative to the LPS group.

To identify the molecular mechanism underlying this effect, the expression levels of iNOS, primarily responsible for NO synthesis, and COX-2, a key pro-inflammatory enzyme that is activated by LPS [21, 22], were quantified. Western blot analysis showed significantly upregulated iNOS and COX-2 expression in LPS-stimulated cells relative to untreated control cells, while pretreatment with CC-LS dose-dependently downregulated the expression of these proteins (Fig. 2A). These findings indicate that the inhibitory effect of CC-LS on NO production was mechanistically associated with iNOS downregulation and that CC-LS generally suppressed inflammatory enzyme expression by inhibiting COX-2 (Fig. 2B and 2C). Overall, these findings indicate that the inhibitory effect of CC-LS on NO production may be due to the downregulation of the expression levels of iNOS and COX-2 rather than cytotoxic effects.

### CC-LS Attenuated Intracellular ROS Generation in LPS-Stimulated RAW 264.7 Cells

**Fig. 3:**
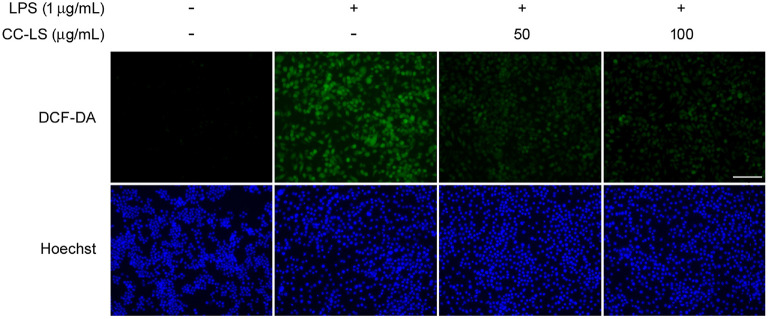
Effect of CC-LS on intracellular ROS production in activated macrophages. Cells were pretreated with CC-LS for 24 h and then stimulated in the absence or presence of LPS (1 μg/ml) for 24 h. ROS production was then visualized via fluorescence microscopy using the cell-permeant dye, DCF-DA. Hoechst was used as a fluorescent marker for the nucleus (Scale bars = 100 μm).

ROS overproduction is a key pathogenic event that occurs during macrophage-mediated inflammatory activation [23, 24]. To determine the effect of CC-LS extract treatment on oxidative stress in activated macrophages, intracellular ROS levels were determined using DCF-DA fluorescence staining. Thus, we observed that LPS-stimulated cells showed significantly higher intracellular ROS levels than untreated control cells. However, pretreatment with CC-LS significantly attenuated LPS-induced ROS accumulation in a concentration-dependent manner (Fig. 3, upper panels). Further, Hoechst nuclear staining confirmed comparable cell density across groups, excluding the possibility that cell loss accounted for differences in the ROS accumulation (Fig. 3, bottom panels). These findings indicate that CC-LS mitigated oxidative stress during macrophage activation and further suggest that the suppression of ROS generation may represent an additional mechanism that contributes to its anti-inflammatory effect.

### CC-LS Inhibited NF-κB Activation and Downregulated Pro-Inflammatory Cytokine Expression

**Fig. 4:**
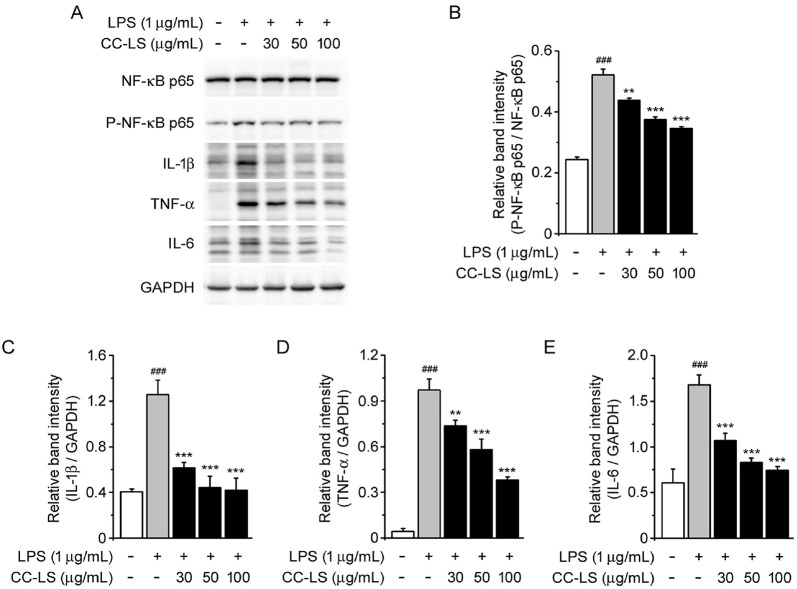
Effect of CC-LS on NF-κB signaling and expression. (**A**) Western blot bands for NF-κB p65, P-NF-κB p65, IL- 1β, TNF-α, and IL-6 expression, with GAPDH as the loading control. (**B**) Band intensity for P-NF- κB p65 relative to NF- κB p65. (**C**) Band intensity for IL-1β relative to GAPDH. (**D**) Band intensity for TNF- α relative to GAPDH. (**E**) Band intensity for IL-6 relative to GAPDH. The values in the bar graphs represent the mean ± SEM of at least three independent experiments. ^###^*P* < 0.005, significant difference relative to the control group; *P < 0.05, **P < 0.01, ***P < 0.005, significant differences relative to the LPS group.

NF-κB signaling is a major pathway that regulates the transcription of inflammatory mediators in macrophages [10]. To determine whether CC-LS extract regulates this pathway, the phosphorylation status of NF-κB p65 was examined via immunoblotting. Thus, we observed that LPS stimulation markedly increased the level of phosphorylated p65 relative to total p65, implying NF-κB activation [25, 26]. However, CC-LS pretreatment attenuated this effect of LPS in a dose-dependent manner (Fig. 4A and 4B).

To investigate the downstream consequences of this inhibitory effect of CC-LS on NF-κB, the protein expression levels of major pro-inflammatory cytokines were determined. The results thus obtained indicated that LPS stimulation significantly increased the levels of IL-1β, TNF-α, and IL-6 in the LPS-treated cells relative to their levels in the control cells. However, CC-LS pretreatment significantly suppressed the expression of these three cytokines (Fig. 4A and 4C–4E). suggesting that this plant extract not only inhibits NF-κB activation, but also attenuates the expression of pro-inflammatory cytokines, thereby preventing LPS-induced inflammatory responses.

### CC-LS Attenuated MAPK Signaling Cascades in LPS-Stimulated Macrophages

**Fig. 5:**
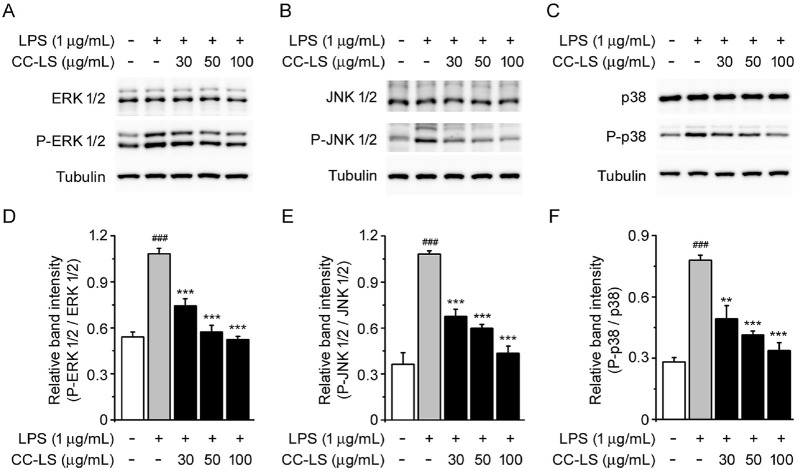
Effect of CC-LS on MAPK signaling pathways in LPS-stimulated macrophages. Western blotting analysis of: (**A**) ERK 1/2 and phosphorylated ERK 1/2 (P-ERK 1/2) expression, (**B**) JNK 1/2 and P-JNK 1/2 expression, and (**C**) p38 and P-p38 expression, with Tubulin as the loading control. (**D**) Level of P-ERK 1/2 quantified via densitometry and normalized to that of ERK 1/2. (**E**) Level of P-JNK 1/2 relative to that of JNK 1/2. (**F**) Level of P-p38 relative to that of p38 ratio. The values in the bar graphs represent the mean ± SEM of at least three independent experiments. ^###^*P* < 0.005, significant difference relative to the control group; *P < 0.05, **P < 0.01, and ***P < 0.005, significant differences relative to the LPS group.

To further investigate the molecular mechanism underlying the anti-inflammatory effects of CC-LS extract, the MAPK signaling pathway was investigated. Specifically, MAPKs, including ERK, JNK, and p38, which are critical mediators of LPS-induced signal transduction, linking extracellular stimuli to pro-inflammatory mediator production in macrophages [12], were quantified via immunoblot analysis, which showed significant increases in the phosphorylation levels of these three kinases following LPS stimulation. This observation is indicative of the robust activation of the MAPK pathway. In contrast, CC-LS pretreatment inhibited the phosphorylation of these three kinases in a concentration-dependent manner, with the total levels of ERK, JNK, and p38 proteins remaining unchanged relative to the untreated control cells (Fig. 5A–5F). These findings suggest that CC-LS suppressed LPS-induced MAPK activation, thereby limiting the propagation of downstream signals that drive pro-inflammatory mediator expression.

### CC-LS Activated AMPK Signaling under Basal Conditions

**Fig. 6:**
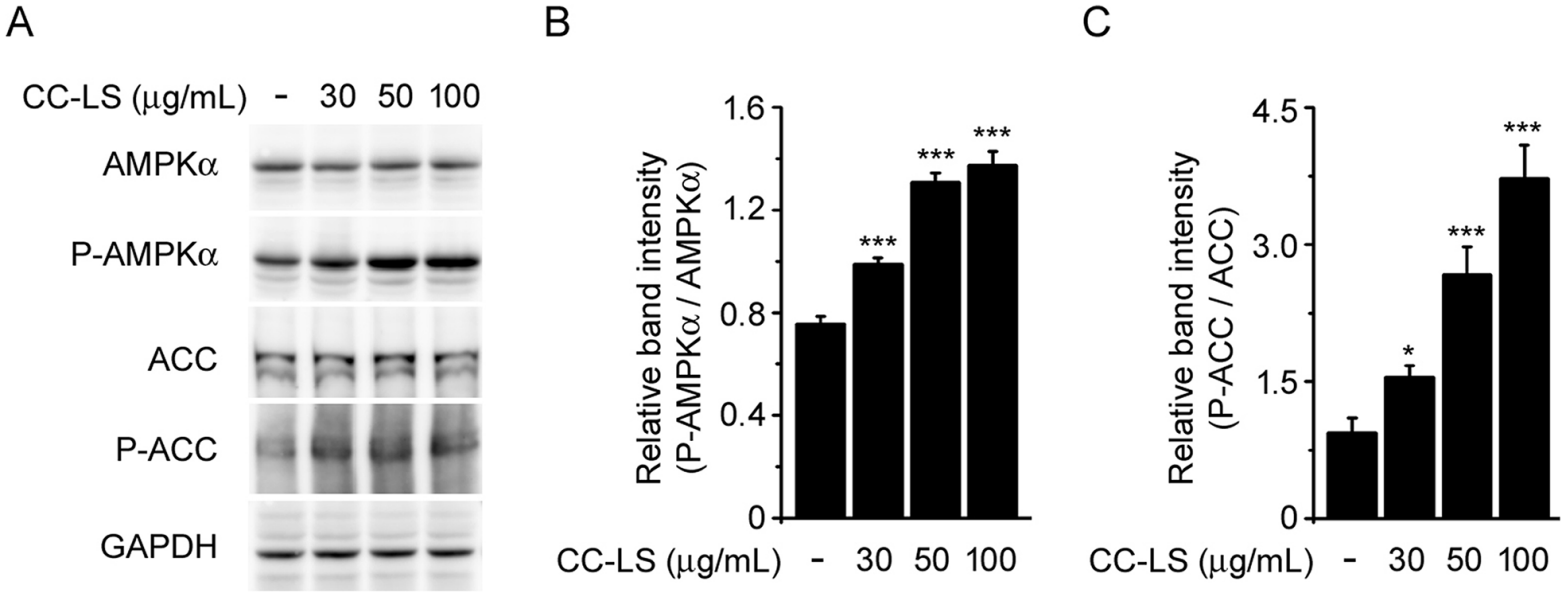
Effects of CC-LS on AMPK signaling under basal conditions. RAW 264.7 cells were treated with different concentrations of CC-LS for 18 h. Thereafter, cell lysates were analyzed via western blot analysis. (**A**) Representative western blots of endogenous AMPKα, P-AMPKα, ACC, P-ACC, and GAPDH, with GAPDH as the loading control. (**B**) Relative band intensity of P-AMPKα quantified via densitometric analysis and normalized to that of total AMPKα. (**C**) Relative band intensity of P-ACC quantified using densitometric analysis and normalized to that of total ACC. The values in the bar graphs represent the mean ± SEM of at least three independent experiments. ^###^*P* < 0.005, significant difference relative to the control group; *P < 0.05, **P < 0.01, and ***P < 0.005, significant differences relative to the control group.

AMPK is a central regulator of cellular energy metabolism [27, 28] and has been shown to negatively regulate inflammatory responses via NF-κB signaling modulation [29, 30]. To further elucidate the molecular basis of the anti-inflammatory effects of CC-LS extract in LPS-stimulated macrophages and identify potential upstream pathways, we examined its effects on AMPK activation. Thus, the level of AMPK activation based on the ratio of phosphorylated AMPKα (Thr172) to total AMPKα showed that CC-LS pretreatment enhanced AMPK phosphorylation in a concentration-dependent manner (Fig. 6A and 6B) while also enhancing the phosphorylation of acetyl-CoA carboxylase (ACC), a well-characterized downstream substrate of AMPK (Fig. 6A and 6C). These findings indicate that the anti-inflammatory effect of CC-LS is mediated, at least in part, via the activation of the AMPK signaling pathway.

## Discussion

Inflammation is a fundamental immune response that protects host cells and tissues against infection and injury. However, when excessive or uncontrolled, it contributes to the pathogenesis of various chronic disorders, including arthritis, cardiovascular disease, and cancer [3-5]. Activated macrophages are particularly important in the inflammatory response as they release pro-inflammatory mediators, such as NO, ROS, and cytokines (*e.g.*, TNF-α, IL-6, and IL-1β) during immune defense [31, 32]. The overproduction of these inflammatory factors not only disrupts tissue homeostasis but also exacerbates inflammatory pathology [33, 34]. Consequently, regulating macrophage-derived mediators has been recognized as a promising strategy for developing novel anti-inflammatory agents. Additionally, natural products have emerged as suitable candidates for developing such drugs owing to their diverse bioactivities and relatively low toxicities [35, 36].

In this study, we showed that CC-LS extract exerted significant anti-inflammatory activity in LPS-stimulated RAW 264.7 macrophages. Notably, CC-LS markedly inhibited LPS-induced NO production without affecting cell viability, confirming that its effect in this regard was not due to cytotoxicity. CC-LS also downregulated the expression of iNOS and COX-2, which are critical indicators of inflammatory activation in macrophages. It also suppressed intracellular ROS generation, suggesting that it offers protection against inflammation-associated oxidative stress and the modulation of both enzymatic and oxidative mediators of macrophage activation.

Our findings further demonstrated that CC-LS regulates multiple intracellular signaling pathways that drive inflammatory responses. NF-κB, a key transcription factor that governs the expression of inflammatory mediators and cytokines, plays a critical role in inflammatory response regulation [10]. In this study, CC-LS significantly attenuated the phosphorylation of p65, which serves as a direct indicator of NF-κB activation. This inhibitory effect was accompanied by decreases in the levels of pro-inflammatory cytokines, including IL-1β, TNF-α, and IL-6, which are not only biomarkers of inflammation but also contribute significantly to disease progression [31-34]. Specifically, IL-1β, a member of the IL-1 family, is strongly associated with innate immunity and triggers autoinflammatory processes [37], and TNF-α, primarily produced by macrophages and other immune cells, exerts broad pro-inflammatory effects that include enhancing leukocyte recruitment, stimulating additional cytokine release, and modulating cell survival and apoptosis [38]. Further, IL-6 promotes inflammation and worsens the acquired immune response by promoting antibody production and T cell differentiation [39, 40]. By suppressing p65 phosphorylation and the downstream release of these cytokines, CC-LS interfered with a key regulatory pathway that drives macrophage-mediated inflammation.

In parallel, CC-LS attenuated the phosphorylation of MAPKs, including ERK, JNK, and p38, which are well-known regulators of cytokine production and inflammatory gene expression [12, 13]. Therefore, the inhibition of MAPK phosphorylation by CC-LS complemented its suppression of NF-κB, suggesting the coordinated regulation of pro-inflammatory signaling.

Furthermore, CC-LS activated AMPK, a serine/threonine kinase that not only functions as a cellular energy sensor, but also serves as an important anti-inflammatory regulator [41-43]. Reportedly, AMPK activation suppresses NF-κB and MAPK signaling pathways, thereby reducing the transcription of pro-inflammatory genes [44-46]. In this study, CC-LS not only promoted AMPK phosphorylation but also enhanced the phosphorylation of its downstream substrate ACC, indicating that the extract functionally activated the AMPK pathway. These findings provide mechanistic evidence that CC-LS utilizes this pathway to attenuate inflammatory signaling.

Therefore, CC-LS exerts broad spectrum anti-inflammatory effects in macrophages by suppressing NO production, downregulating iNOS and COX-2 expression, inhibiting ROS generation as well as NF-κB and MAPK signaling, and activating AMPK. This multifaceted regulatory effect supports CC-LS extract as a promising candidate for developing anti-inflammatory agents.

To the best of our knowledge, this study is the first systematic report describing the anti-inflammatory effects of CC-LS extract in macrophages, while also highlighting the underlying mechanisms. Additionally, the findings of this study enhance current understanding regarding the plants of the genus *Cornus* and support further investigations on CC-LS as a potential anti-inflammatory agent. In future studies, it would also be necessary to identify the active constituents responsible for these effects and validate the findings reported here using appropriate *in vivo* models of inflammation.

In conclusion, CC-LS significantly attenuated LPS-induced inflammatory responses in macrophages by inhibiting iNOS and COX-2 expression, ROS production, and cytokine production, while also suppressing NF-κB and MAPK pathways and activating AMPK. These findings may serve as a basis for using CC-LS as a natural extract with potential for the development of anti-inflammatory agents.
